# Invited commentary: nutrition during growth and reproduction: studies demonstrating possibilities and difficulties

**DOI:** 10.3402/gha.v7.23484

**Published:** 2014-01-03

**Authors:** Elisabet Forsum

**Affiliations:** Department of Clinical and Experimental Medicine, Linköping University, Linköping, Sweden

Commentary on a thesis by Ashraful Islam Khan ‘Effects of Pre- and Postnatal Nutrition Interventions on Child Growth and Body Composition’.

Ashraful Islam Khan has written a thesis that provides important and interesting aspects on several issues relevant for the nutritional situation of reproductive women and young children. Dr. Khan's work forms a part of the ‘MINIMat’ (Maternal and Infant Nutrition Interventions in Matlab) trial in rural Bangladesh. This trial is a population-based randomized food and micronutrient intervention where pregnant women were randomly allocated to an early or usual timing of invitation to food supplementation, which included three different micronutrient alternatives. This was coupled with exclusive breastfeeding counseling or with the health message commonly directed at childbearing women in this population. Results obtained in the MINIMat study have previously been published (1–5) and the findings include data regarding micronutrient status in women and children as well as important effects of micronutrient and food supplementation on offspring birth weight and mortality. Dr. Khan's thesis is focused on the effects of prenatal nutritional supplementation and the type of breastfeeding counseling on physical growth and body composition development of the offspring. All studies in the thesis are carried out in Matlab, a rural district situated in a river delta southeast of Dhaka. The area has a population of about 220,000 people, and the International Centre for Diarrhoeal Disease Research has recorded health and demographic information there since 1966. They also provide health centers serving the population in the area with services such as family planning counseling and basic healthcare.

The first paper in the thesis (6) is a randomized trial investigating the effects of prenatal food and micronutrient supplementation on child growth from birth to 54 months of age. A total of 4,436 pregnant women were enrolled in the study for comparing nutritional supplementation starting very early versus rather late in gestation. The supplement provided 2,500 kJ 6 days a week. Three different micronutrient supplements, two combinations of iron and folate and a third containing 15 micronutrients, including iron and folate, were also tested and the women were therefore divided into six groups. Interesting findings were that early supplementation reduced the occurrence of stunting in boys but not in girls, and also that prenatal multiple micronutrient supplementation increased the proportion of stunted boys. Findings partly along this line have been presented earlier, for example Gibson (7) reviewed double-blind zinc supplementation studies in children and reported that improved growth was more commonly observed in males than in females. These results and the findings by Dr. Kahn may have a common explanation, i.e. that boys have higher requirements than girls for essential nutrients simply because they are bigger and gain more weight and length during infancy and childhood. The observation that prenatal multiple micronutrient supplementation increased the proportion of stunted boys is important and intriguing. It can be reconciled with a number of observations showing that providing nutrients as supplements to humans may sometimes have detrimental effects. This fact has been emphasized in the recent issue of nutritional recommendations for the Nordic countries (8). With respect to pregnant women, there is one well-known example from a study in a poor black population in New York City where protein supplementation in women during pregnancy increased the proportion of premature births and associated neonatal deaths, as well as the number of growth-retarded fetuses (9).

An important goal in the MINIMat trial was to study the appropriate timing of food supplementation to pregnant women. Results reported by Ceesay et al. (10), as well as results from the Dutch hunger winter (11), seem to indicate that fetal growth is most sensitive to nutritional deprivation during the last trimester of pregnancy. However, the findings by Kahn et al. (6) suggest that providing a supplement immediately after the identification of pregnancy (usually around gestational week nine), rather than in the second semester, resulted in less stunting in male offspring. Although these results need confirmation, they are indeed interesting since they demonstrate how limited our understanding is when it comes to fundamental aspects on how nutrition influences human reproduction and the consequences such influences have for offspring development and health. In relation to the studies conducted by Dr. Kahn, it is certainly important to point out that improved nutrition of reproductive women has been shown to decrease plasma prolactin (12), thereby shortening the duration of post-partum amenorrhea. It is therefore appropriate to note that, in the Matlab area, female community health workers visited each household every month providing family planning counseling and contraceptive health service.

Another important part of the thesis is the ambition to study not only the growth of children born in the study but also their body composition at 54 months of age. This is a very difficult task since few body composition methods are applicable in this age group. Therefore, a study was undertaken to develop a method suitable for the children in the study (13). For this purpose, a leg-to-leg bioelectrical impedance analyzer (Tanita) was used at a frequency of 50 kHz. A total of 200 children, aged 4–10 years, were given a dose of deuterium oxide to assess their total body water and based on this estimate their body composition, that is, their fat mass as well as their fat-free mass, was calculated. This is a well-established method used to assess body composition in children, which certainly is acceptable as a reference method. Using these reference estimates of fat-free mass, an equation was developed for assessing this variable based on values obtained by means of the bioelectrical impedance analyzer. It was found that the output of this analyzer could, together with weight, height, age, and sex, explain 89% of the variation in the fat-free mass of the children. This equation was considered satisfactory and was used to assess body composition of the children in the study at 54 months of age (14). A very wise decision was to also include measurements of skinfolds. In this way, two independent estimates of the children's body composition were obtained. None of them gave any indication that body composition was different in any of the six subgroups of the study. Therefore, it seems very likely that this is the correct result. Without skinfold data this conclusion would have been more difficult. In fact, there are a number of publications reporting the validation of the bioelectrical impedance method in children. Thus, Talma et al. (15) published a systematic review of such studies, conducted in children aged 5–18 years, excluding studies of poor quality. On the basis of their findings, they concluded that the validity and measurement error of the bioelectrical impedance method is unsatisfactory for children and adolescents. It is certainly true that their review did not include data from 4-year olds and the contribution by Kahn et al. (13), which provides such data, should indeed be recognized. However, it is also important to recognize that a validation study of their impedance-based equation is not yet available.

The final part of the thesis deals with breastfeeding and investigates if exclusive breastfeeding counseling is superior to a ‘usual health message’ in promoting lactation. Khan et al. (16) reported that women who received exclusive breastfeeding counseling breastfed for 35 more days than mothers who received the usual health message. This is encouraging since it demonstrates that intensive counseling may have the capacity to increase the duration of lactation. Obviously, this is of great value, especially in a low-income country such as Bangladesh, for the health and development of young children. Furthermore, as discussed in this thesis, breastfeeding is likely to have additional long-term effects on health that may be more difficult to assess. After the thesis was presented, a systematic literature review on the topic has been published (17). This review concluded that there is convincing evidence of a protective effect of breastfeeding on overweight and obesity in childhood and adolescence, on overall rate of infections, acute otitis media, and gastrointestinal and respiratory tract infections. In addition, this review (17) identified probable evidence for a protective effect of breastfeeding on inflammatory bowel disease, celiac disease and diabetes types 1 and 2 as well as for a small reductive effect on blood pressure and blood cholesterol levels in adulthood. These findings seem to provide ample motivation for all possible attempts to support and increase breastfeeding in all kinds of populations.

The studies in Dr. Khan's thesis demonstrate different aspects on the current knowledge regarding nutrition during reproduction and growth. They show that we already have much of the knowledge required to improve the situation for malnourished women, infants, and children. Apparently, supplementing women during pregnancy and counseling them regarding the importance of breastfeeding can make a considerable difference. On the contrary, important gaps of knowledge remain and this thesis has indeed contributed to identify such areas. This kind of contribution is also very important since this is the way new knowledge can be gained.
